# ELYS regulates the localization of LBR by modulating its phosphorylation state

**DOI:** 10.1242/jcs.190678

**Published:** 2016-11-15

**Authors:** Yasuhiro Mimura, Masatoshi Takagi, Michaela Clever, Naoko Imamoto

**Affiliations:** Cellular Dynamics Laboratory, Riken, Saitama 351-0198, Japan

**Keywords:** Nuclear envelope, Nuclear pore complex, Inner nuclear membrane protein, Nucleoporin, Phosphorylation

## Abstract

Lamin B receptor (LBR), an inner nuclear membrane (INM) protein, contributes to the functional integrity of the nucleus by tethering heterochromatin to the nuclear envelope. We have previously reported that the depletion of embryonic large molecule derived from yolk sac (ELYS; also known as AHCTF1), a component of the nuclear pore complex, from cells perturbs the localization of LBR to the INM, but little is known about the underlying molecular mechanism. In this study, we found that the depletion of ELYS promoted LBR phosphorylation at the residues known to be phosphorylated by cyclin-dependent kinase (CDK) and serine/arginine protein kinases 1 and 2 (SRPK1 and SRPK2, respectively). These phosphorylation events were most likely to be counter-balanced by protein phosphatase 1 (PP1), and the depletion of PP1 from cells consistently caused the mislocalization of LBR. These observations point to a new mechanism regulating the localization of LBR, which is governed by an ELYS-mediated phosphorylation network. This phosphorylation-dependent coordination between INM proteins and the nuclear pore complex might be important for the integrity of the nucleus.

## Abstract (precis)

**Highlighted Article:** The nuclear envelope localization of LBR is regulated through a phosphorylation network governed by ELYS, and defects in this phosphorylation network induce the aberrant phosphorylation and mislocalization of LBR.

## INTRODUCTION

The nuclear envelope, which encloses the eukaryotic genome, is composed of double lipid bilayers termed the outer nuclear membrane (ONM) and the inner nuclear membrane (INM). The ONM connects to the endoplasmic reticulum (ER), whereas the INM contains a specific set of transmembrane proteins termed INM proteins. The nuclear lamina, which is composed of A-type and B-type lamins, is located underneath the INM and provides mechanical strength to the nuclear envelope. Another characteristic of the nuclear envelope is the presence of nuclear pore complexes (NPCs) that perforate the nuclear envelope at the sites where the INM and ONM fuse (Hetzer et al., 2005). These peripheral nuclear structures are important structures to which heterochromatin is tethered and through which vital biological processes, such as transcription and genome stability, are regulated (Mekhail and Moazed, 2010).

Lamin B receptor (LBR), an INM protein, has crucial roles, including the tethering of heterochromatin to the nuclear periphery (Solovei et al., 2013), chromatin compaction and transcriptional repression (Hirano et al., 2012). Thus, LBR is considered a key component in the establishment of the heterochromatic environment of the nuclear periphery.

The NPC, which comprises multiple copies of ∼30 distinct proteins termed nucleoporins (Nups), is the solitary gateway for bi-directional macromolecular transport between the cytoplasm and the nucleus (D'Angelo and Hetzer, 2008). The NPC comprises two types of gateways – a central channel and a peripheral channel (also called a lateral channel). Soluble macromolecules pass through the central channel, typically with the assistance of nuclear transport receptors (Kimura and Imamoto, 2014). In contrast, the transmembrane-domain-containing INM proteins pass through the peripheral channel (Katta et al., 2014). To become localized in the INM after synthesis in the ER, LBR must traverse the peripheral channel, which requires the function of Nup and defined elements within the LBR.

LBR is composed of an N-terminal nucleoplasmic region, which acts as the interface for interactions with many binding partners, followed by eight putative transmembrane regions, which have homology with cholesterol reductases such as human TM7SF2 and DHCR7 (Olins et al., 2010). The N-terminal region of LBR comprises a Tudor domain (also called the globular I domain) and a globular II domain, which are linked by a hinge region (Liokatis et al., 2012; Ye et al., 1997). The hinge region contains a nuclear localization signal (NLS), which is required for the interaction with importin β (Ma et al., 2007), and an arginine-serine repeat (RS) domain, which consists of multiple repeats of arginine and serine residues (Sellis et al., 2012). The entire N-terminal region is required for interaction with lamin B (Ye and Worman, 1994). Both the Tudor and RS domains interact with DNA (Duband-Goulet and Courvalin, 2000; Ye and Worman, 1994) and core histones (Makatsori et al., 2004; Polioudaki et al., 2001; Takano et al., 2002), and the globular II domain interacts with heterochromatin protein-1 (HP-1; also known as CBX1) (Ye et al., 1997; Ye and Worman, 1996). Additionally, the Tudor domain interacts with specific histone modifications, such as histone H4 K20 di-methylation, and this ability to bind to modified histones is important for the nuclear envelope localization of LBR (Hirano et al., 2012).

Previous *in vitro* assays have shown that LBR is phosphorylated at residues S71 and S86 by cyclin-dependent kinase (CDK) (Lu et al., 2010; Nikolakaki et al., 1997; Tseng and Chen, 2011), and serine residues within the RS domain are phosphorylated by serine/arginine protein kinases 1 and 2 (SRPK1 and SRPK2, respectively) (Nikolakaki et al., 1997, 1996; Sellis et al., 2012; Tsianou et al., 2009). However, phosphorylation at these sites is removed by the γ1 isoform of protein serine/threonine phosphatase-1 (PP1γ1) (Ito et al., 2007). LBR phosphorylation promotes many of the interactions with the binding partners described above (Appelbaum et al., 1990; Lu et al., 2010; Takano et al., 2004, 2002).

Embryonic large molecule derived from yolk sac (ELYS; also known as AHCTF1) is a chromatin-binding nucleoporin that possesses an AT-hook domain at its C-terminus. This protein plays an initial role in post-mitotic NPC assembly (Doucet et al., 2010; Galy et al., 2006; Inoue and Zhang, 2014; Rasala et al., 2006; Zierhut et al., 2014). We have previously reported that the depletion of ELYS perturbs the recruitment of LBR to the reforming nuclear envelope during telophase (Clever et al., 2012). In the present study, we found that the nuclear envelope localization of LBR is also impaired in interphase upon depletion of ELYS. Therefore, ELYS is a key determinant of the nuclear envelope localization of LBR throughout the cell cycle.

Here, we show that ELYS regulates the nuclear envelope localization of LBR in interphase by modulating its phosphorylation status. ELYS depletion promoted the phosphorylation of LBR at residues S71, S86 and serine residues within the RS domain. Phosphomimetic mutations at those sites were sufficient to reduce the nuclear envelope localization of LBR. Interestingly, the depletion of NUP107 and NUP153 also induced defects in the localization and phosphorylation state of LBR that were similar to those observed in response to ELYS depletion, implying that a phosphorylation network governed by NPC components might exist and regulate the integrity of the nucleus through the modulation of LBR.

## RESULTS

### Nuclear envelope localization and LBR phosphorylation are impaired upon ELYS depletion

**Fig. 1.**
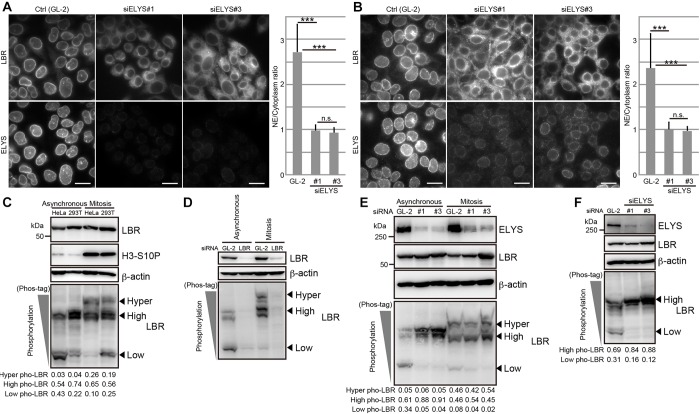
**ELYS depletion induces LBR mislocalization and phosphorylation.** HeLa (A) and HEK293T (B) cells were transfected with control (Gl-2, control siRNA against luciferase) or siRNAs against ELYS (siELYS#1 or siELYS#3), and then cultured for 48 h. The subcellular localization of LBR and ELYS was observed by immunostaining. The ratios of LBR staining intensities in the nuclear envelope (NE) to those in the cytoplasm were calculated (see Materials and Methods) and plotted. The calculated nuclear envelope to cytoplasm ratios were analyzed by using an unpaired Student's *t*-test; error bars, s.d.; n.s., not significant; ***P<0.001 (*n*=30). Scale bars: 20 µm. (C) Phosphorylation of LBR in asynchronous or mitotically arrested HeLa and HEK293T cells. The phosphorylation of LBR was analyzed with Phos-tag western blotting. (D) HeLa cells that had been transfected with either control (GL-2) or an siRNA against LBR (siLBR) were cultured for 48 h (Asynchronous) or cultured for 32 h and then treated with nocodazole for 16 h (Mitosis). The phosphorylation of LBR was analyzed using Phos-tag western blot. The relative amount of LBR was ∼20% of that in control cells in both the asynchronous and the mitotic cells. (E) HeLa cells that had been transfected with the indicated siRNAs against ELYS were cultured for 48 h (Asynchronous). The cells, which were transfected with the same siRNAs as in the asynchronous condition, were treated with nocodazole combined with a double thymidine block to arrest the cells at mitosis. The phosphorylation of LBR was analyzed by using Phos-tag western blotting. The relative amount of ELYS was ∼20% of that in control cells under every indicated condition. (F) HEK293T cells were transfected with the control (GL-2), siELYS#1 or siELYS#3 and cultured for 48 h. The harvested cells were analyzed with Phos-tag western blotting. The relative amount of ELYS was ∼20% of that in control cells in the siELYS#1-transfected cells and ∼10% of that in control cells in the siELYS#2-transfected cells. In C,E,F, the arrowheads indicate the positions of the different phosphorylated forms of LBR (hyper, high and low levels of phosphorylation). The signal intensities of the different phosphorylated forms of LBR relative to those in total signal intensities (Hyper, High and Low) are shown below the Phos-tag images. Pho, phosphorylation; H3-S10P, phosphorylated histone H3 S10, mitotic marker; β-actin, loading control.

We have reported previously that ELYS is required for the accumulation of LBR in the reforming nuclear envelope at the end of mitosis (Clever et al., 2012). In this study, we noticed that ELYS is also required for the interphase localization of LBR. Although LBR was primarily confined to the nuclear envelope in control cells, it became dispersed throughout the ER and was not restricted to the nuclear envelope in cells that had been treated with an ELYS-specific siRNA (Fig. 1A,B; Fig. S1A). Essentially the same observations were obtained in two different cell lines (HeLa and HEK293T cells) with two different siRNAs (siELYS#1 or siELYS#3) (Fig. 1A,B; Fig. S1A), supporting the generality and the reproducibility of the effects.

We next asked whether ELYS depletion affected the subcellular localization of other nuclear envelope components. The nuclear envelope localization of lamin A/C (encoded by *LMNA*), lamin B, emerin and Lap2α and Lap2β (two isoforms encoded by *TMPO* through alternative splicing) were not obviously affected by ELYS depletion (Fig. S1B–E), although cytoplasmic aggregates of lamin B, emerin and Lap2β were observed in some cells (Fig. S1B and D, arrows).

ELYS depletion severely perturbs NPC assembly in post-mitosis cells (Doucet et al., 2010; Galy et al., 2006; Rasala et al., 2006) (Figs S1F, S2A, S4). We assessed the nuclear transport activity of the ELYS-depleted cells using a reporter cargo containing the SV40T antigen NLS (mCherry–NLS). The cargo efficiently accumulated in the nucleus to an extent similar to that observed in control (Fig. S1G), suggesting the central channel of NPC retained functionality in the ELYS-depleted cells.

The nuclear envelope localization of LBR is established through interactions between its N-terminus and many binding partners (Hirano et al., 2012; Lu et al., 2010), and many of these interactions are regulated by CDK- and SRPK-mediated LBR phosphorylation (Appelbaum et al., 1990; Takano et al., 2004, 2002). Thus, we predicted that the nuclear envelope localization of LBR is regulated by phosphorylation. To analyze the phosphorylation status of LBR, we used Mn^2+^-Phos-tag western blotting. Phos-tag is a chemical reagent that specifically binds to the phosphate group and retards the mobility of phosphorylated proteins on SDS-PAGE gels (Kinoshita-Kikuta et al., 2007). LBR shows at least two different phosphorylated forms during interphase (Fig. 1C, asynchronous); the form with lower levels of phosphorylation is represented by high-mobility protein bands on Phos-tag gels, and the form with higher levels of phosphorylation is represented by low-mobility protein bands on the gels. During mitosis, a hyper-phosphorylated form of LBR was also observed, as shown by the retarded mobility of the LBR bands on the Phos-tag gel (Fig. 1C, Mitosis). All of these bands were lost when the cells were treated with an siRNA against LBR (Fig. 1D), confirming the specificity for the LBR phosphorylation. When examined in the ELYS-depleted cells, we found that the level of LBR phosphorylation was substantially elevated in asynchronous HeLa and HEK293T cells (Fig. 1E,F) but not in mitotic HeLa cells (Fig. 1E).

Taken together, these results indicate that ELYS depletion not only induces the mislocalization of LBR from the nuclear envelope but also promotes LBR phosphorylation. We presumed that there was a causal relationship between these two events.

### Depletion of NUP107 or NUP153, but not of POM121, also induces the mislocalization and aberrant phosphorylation of LBR

**Fig. 2.**
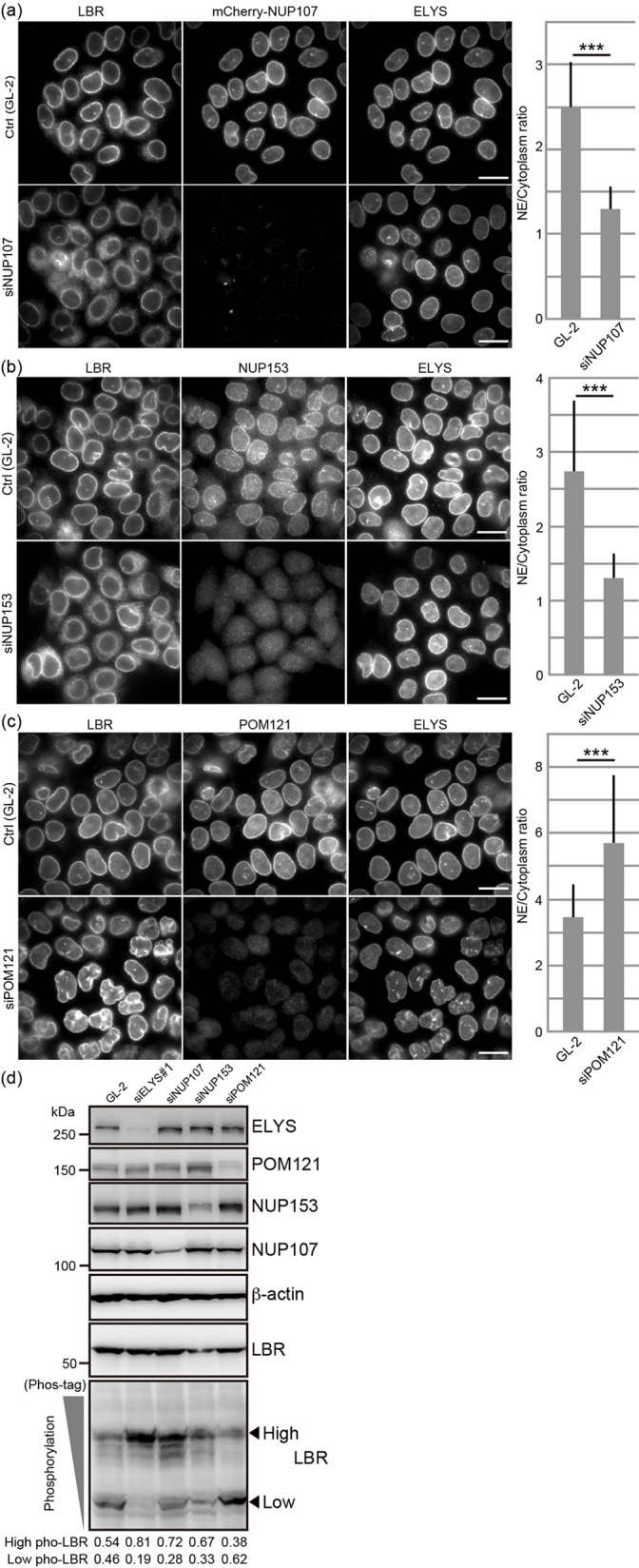
**Depletion of NUP107 or NUP153, but not POM121, induces the mislocalization and phosphorylation of LBR.** HeLa cells were transfected with siRNAs against NUP107 (A, siNUP107), NUP153 (B, siNUP153) and POM121 (C, siPOM121) and cultured for 48 h, after which the subcellular localization of LBR was observed. Scale bars: 20 µm. Quantification of the ratios of LBR staining intensities in the nuclear envelope (NE) to those in the cytoplasm are shown on the right. Error bars, s.d.; ***P<0.001 (unpaired Student's *t*-test). (D) HeLa cells were transfected with the siRNA against ELYS (siELYS#1) and cultured for 48 h, after which the phosphorylation status of LBR was analyzed by Phos-tag western blotting. The arrowheads indicate the positions of signals for the different phosphorylated forms of LBR (high and low levels of phosphorylation, as indicated). The signal intensities of high and low phosphorylation forms of LBR relative to total signal intensities are shown below the image. Pho, phosphorylation. The relative amount of each Nup was ∼10% of that in control cells in the siELYS#1-transfected cells, ∼50% of that in control cells in the siPOM121-transfected cells, ∼40% of that in control cells in the siNUP153-transfected cells and ∼60% of that in control cells in the siNUP107-transfected cells. β-actin, loading control.

We investigated if the mislocalization and aberrant phosphorylation of LBR were only caused by ELYS depletion or if they could also be caused by the depletion of other Nups, such as NUP107, NUP153 or POM121. Depletion of NUP107 and NUP153 impaired the nuclear envelope localization of LBR (Fig. 2A,B), whereas depletion of POM121 did not (Fig. 2C). LBR phosphorylation was promoted by the depletion of NUP107 and NUP153 but not by the depletion of POM121 (Fig. 2D). Previous reports have shown that the number of NPCs is a crucial parameter for the targeting of transmembrane proteins to the INM from the ER (Boni et al., 2015; Ungricht et al., 2015). For this reason, we compared the density of foci resulting from staining with mAb414 (recognizing nuclear pore complex proteins, such as NUP358, NUP214, NUP153 and NUP62) between control cells and cells that had been depleted of Nups (Fig. S2A–D). The density of foci was reduced by ∼38.5% in ELYS-depleted cells, 54.6% in NUP107-depleted cells, 57.4% in NUP153-depleted cells and 22.4% in POM121-depleted cells (Fig. S2E). Despite the moderate effect on reducing the density of NPCs as defined by mAb414-stained foci, relative to the other Nup depletions tested, ELYS depletion impaired the localization and phosphorylation of LBR most strongly (compare Fig. 1A and Fig. 2). It is conceivable that the LBR mislocalization induced by Nup depletion correlates well with its increased phosphorylation.

### CDK, SRPKs and PP1 isoforms regulate the phosphorylation state of LBR

**Fig. 3.**
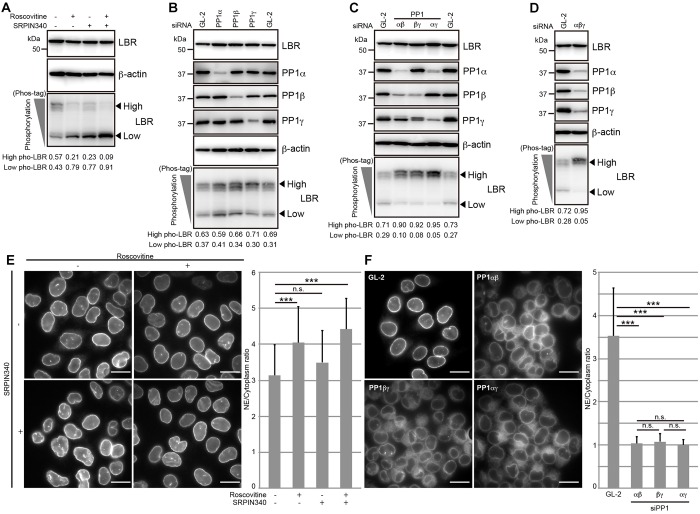
**LBR phosphorylation is regulated by CDK, SRPKs and PP1 isoforms, and the nuclear envelope localization of LBR is impaired by PP1 depletion.** (A) HeLa cells were treated with the indicated inhibitors for 5 h, and then the phosphorylation status of LBR was analyzed with Phos-tag western blotting. (B) HeLa cells were transfected with the indicated siRNAs and then cultured for 48 h. The phosphorylation of LBR was analyzed with Phos-tag western blotting. The relative level of each PP1 isoform was ∼20% of that in control cells in the cells that had been transfected with siRNA against PP1α, ∼30% of that in control cells in the cells that had been transfected with siRNA against PP1β, and ∼30% of that in control cells in the cells that had been transfected with siRNA against PP1γ. (C) HeLa cells were transfected with the indicated combinations of siRNAs against the two PP1 isoforms denoted and then cultured for 48 h. The phosphorylation of LBR was analyzed with Phos-tag western blotting. (D) HeLa cells were transfected with siRNAs against all of the PP1 isoforms and then incubated for 48 h. The phosphorylation of LBR was analyzed with Phos-tag western blotting. (E) HeLa cells were treated with CDK and SRPK inhibitors as described in A, and the subcellular localization of LBR was analyzed. Scale bars: 20 µm. (F) HeLa cells were treated with a combination of siRNAs against the PP1 isoforms as described in C, and the subcellular localization of LBR was analyzed (right-hand panel). Scale bars: 20 µm. In A–D, the arrowheads indicate the positions of the different phosphorylated forms of LBR (high and low levels of phosphorylation, as indicated). The relative signal intensities for the different phosphorylated LBR forms are shown below the Phos-tag images. In E,F, the staining intensities of LBR in the nuclear envelope (NE) relative to those in the cytoplasm were calculated and plotted as described in Fig. 1A. The calculated nuclear envelope to cytoplasm ratios were analyzed using an unpaired Student's *t*-test; error bars, s.d.; n.s., not significant; ***P<0.001 (*n*=30). β-actin, loading control.

Previous *in vitro* phosphorylation and dephosphorylation assays have shown that LBR is phosphorylated by CDK and SRPKs and is dephosphorylated by PP1γ1 at its N-terminal region (Ito et al., 2007; Lu et al., 2010; Nikolakaki et al., 1997, 1996; Papoutsopoulou et al., 1999; Sellis et al., 2012; Takano et al., 2004; Tseng and Chen, 2011; Tsianou et al., 2009). To examine whether LBR phosphorylation is also regulated by CDK and SRPKs in cells, cells were treated with roscovitine, a CDK inhibitor; SRPIN340, an SRPK inhibitor; or both inhibitors, and then the phosphorylation status of LBR was analyzed using Phos-tag western blotting. The amount of the highly phosphorylated form LBR was decreased, whereas the amount of the form of LBR with lower levels of phosphorylation was increased under every condition (Fig. 3A), indicating that LBR phosphorylation is regulated by both CDK and SRPKs in the cells.

Next, we examined the effect of PP1 depletion on LBR phosphorylation status in the cells. Because three PP1 isoforms (PP1s) – PP1α, PP1β and PP1γ (notice that, in mammals, PP1γ has two splicing variants, PP1γ1 and PP1γ2, for which we used PP1γ as the collective term) – are expressed in HeLa cells, we knocked down individual PP1s using an siRNA specific to each isoform. There was no obvious effect on LBR phosphorylation when the individual PP1s were depleted (Fig. 3B). In contrast, when any pair of PP1s was simultaneously knocked down, the level of the highly phosphorylated form of LBR clearly increased (Fig. 3C). When the cells were simultaneously treated with siRNAs against all three PP1s, LBR phosphorylation increased (Fig. 3D). These results indicate that LBR dephosphorylation is regulated by PP1s in a redundant manner and that at least two of the three isoforms are required to achieve the LBR phosphorylation state observed in the control cells.

We next investigated the relationship between the LBR phosphorylation states mediated by the balanced actions of CDK, SRPKs and PP1s and the localization of LBR. LBR phosphorylation was reduced, and the nuclear envelope localization of LBR was maintained in the cells that had been treated with roscovitine, SRPIN340 or both inhibitors (Fig. 3A,E). In contrast, the depletion of any pair of the three PP1s increased LBR phosphorylation and severely impaired the nuclear envelope localization of LBR (Fig. 3C,F). Note that the nuclear envelope localization of LBR was maintained when only one PP1 isoform was knocked down (Fig. S3A). These results suggest that the LBR form with lower levels of phosphorylation can be stably localized to the nuclear envelope.

### LBR phosphorylation at S71, S86 and at serine residues in the RS domain is increased by ELYS depletion, and phosphomimetic mutation of these sites impairs the nuclear envelope localization of LBR

**Fig. 4.**
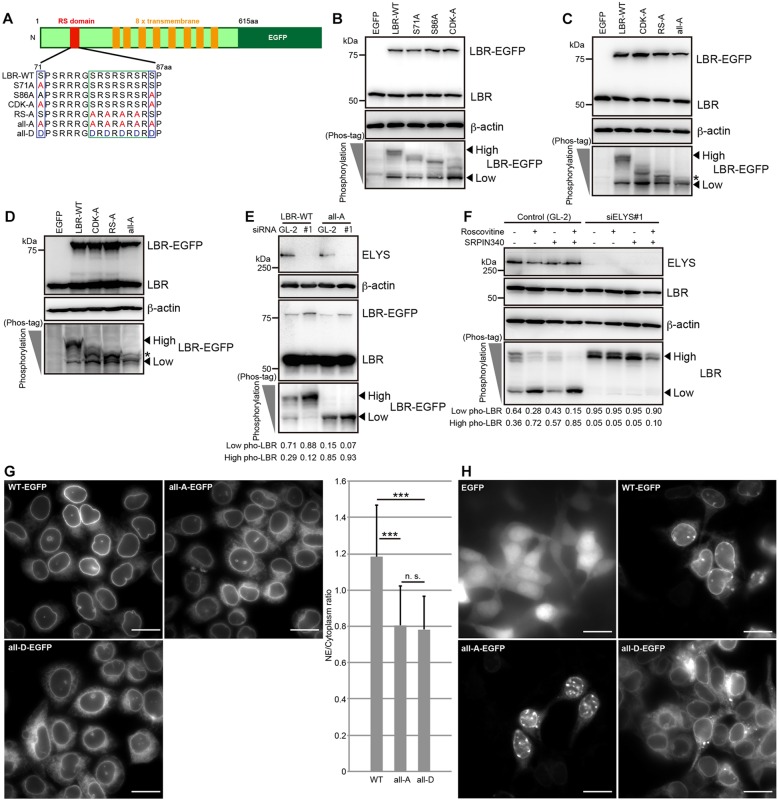
**LBR phosphorylation at S71, S86 and the serine residues within the RS domain is promoted by ELYS depletion, and the nuclear envelope localization of LBR is impaired by phosphomimetic mutations** **at those sites.** (A) Schematic representation of LBR structure and the phosphorylation sites in its N-terminus. The red and orange boxes indicate the RS domain and transmembrane regions, respectively. The given amino acid sequences represent the RS domain and its surrounding region. The serine residues in blue rectangles are phosphorylated by CDK, and the serine residues in the green rectangle are phosphorylated by SRPKs. The S71A and S86A mutants carry alanine substitutions at S71 and S86, respectively. The CDK-A and RS-A mutants carry alanine substitutions at the positions indicated in the blue and green rectangles, respectively. In the all-A and all-D mutants, the serine residues replaced by alanine and aspartic acid residues are indicated with blue and green rectangles, respectively. (B) HeLa cells were transiently transfected with LBR-WT–EGFP or the indicated LBR–EGFP mutants and cultured for 48 h. The phosphorylation of LBR–EGFP was evaluated with Phos-tag western blotting. HeLa (C) and HEK293T (D) cells were transfected with LBR-WT–EGFP or the indicated LBR–EGFP mutants and then cultured for 48 h. The phosphorylation of LBR–EGFP was analyzed with Phos-tag western blotting. The asterisks show the residual phosphorylation signal for LBR-all-A–EGFP. (E) HeLa cells stably expressing LBR-WT–EGFP or LBR-all-A–EGFP were transfected with the indicated siRNAs (GL-2, control; siRNA against ELYS, siELYS#1) and then cultured for 48 h. The phosphorylation of LBR–EGFP was analyzed with Phos-tag western blotting. (F) HeLa cells were transfected with the indicated siRNAs, incubated for 48 h, and further cultured in medium containing the indicated inhibitors for 5 h. LBR phosphorylation was evaluated with Phos-tag western blotting. (G) Subcellular localization of the stably expressed LBR-WT–EGFP, LBR-all-A–EGFP and LBR-all-D–EGFP constructs in HeLa cells. The ratio of the intensity of LBR–EGFP fluorescence at the nuclear envelope (NE) to that at the cytoplasm in these stable cell lines was calculated and plotted. The calculated ratios were analyzed with an unpaired Student's *t*-test; error bars, s.d.; n.s., not significant; ***P<0.001 (*n*=30). Scale bars: 20 µm. (H) Subcellular localization of LBR-WT–EGFP, LBR-all-A–EGFP and LBR-all-D–EGFP proteins that were transiently expressed in HEK293T cells. All of the images were obtained using the same microscope setting. Scale bars: 20 µm. In B–F, the arrowheads indicate the positions of the different phosphorylated forms of LBR (high and low levels of phosphorylation, as indicated). The signal intensities of high and low phosphorylated forms of LBR relative to total signal intensities are shown below the Phos-tag images. β-actin, loading control. WT–EGFP, WT LBR fused to EGFP.

Previous *in vitro* phosphorylation assays have shown that LBR is phosphorylated at S71 and S86 by CDK (Ito et al., 2007; Lu et al., 2010; Tseng and Chen, 2011) and at serine residues within the RS domain by SRPKs (Nikolakaki et al., 1997, 1996; Papoutsopoulou et al., 1999; Sellis et al., 2012; Takano et al., 2004; Tsianou et al., 2009). To investigate whether these serine residues of LBR are phosphorylated in cells, we generated a series of unphosphorylated mutants that are depicted in Fig. 4A. HeLa cells were transfected with EGFP-tagged LBR wild-type (LBR-WT–EGFP) or the unphosphorylated mutants, and then their phosphorylation statuses were analyzed using Phos-tag western blotting. Exogenously expressed LBR-WT–EGFP was phosphorylated as efficiently as endogenous LBR (Fig. 4B compared with Fig. 1C). The S71A–EGFP and S86A–EGFP mutants exhibited increased mobility compared with LBR-WT–EGFP, showing that the phosphorylation of these mutants was reduced (Fig. 4B). The mobility of the CDK-A–EGFP mutant, which harbors both the S71A and S86A mutations, was additively increased, showing that both serine residues are phosphorylated in cells (Fig. 4B). The mobility of the RS-A–EGFP mutant was also increased relative to that of LBR-WT, showing that the residues in the RS domain were also phosphorylated in cells (Fig. 4C). The phosphorylation of the all-A–EGFP mutant containing both the CDK-A and RS-A mutations was appreciably reduced relative to that of LBR-WT, although weak signals just above the form of LBR that had lower levels of phosphorylation were still observed (Fig. 4C, see protein bands indicated by an asterisk). Similar results were also obtained in HEK293T cells (Fig. 4D). Although we cannot exclude the possibility that less-important phosphorylation sites remain, we concluded that S71, S86 and the serine residues within the RS domain are important sites within LBR that are phosphorylated during interphase. The effects on the localization (Fig. 4E) and phosphorylation (Fig. S3D) of LBR-WT–EGFP as a result of ELYS depletion were similar to those on endogenous LBR (Figs 1E and 2D). ELYS depletion did not cause a mobility shift of the all-A–EGFP construct (Fig. 4E), indicating that the serine residues that had been mutated in this construct (Fig. 4A) were the phosphorylation sites that were affected by ELYS depletion.

ELYS depletion could enhance the phosphorylation of LBR either by upregulating CDK and SRPKs or by suppressing PP1s. To distinguish between these two possibilities, the cells were first treated with an ELYS-specific siRNA for 48 h and then treated with CDK and SRPKs inhibitors for an additional 5 h (Fig. 4F). LBR became highly phosphorylated following ELYS depletion, and this effect was maintained even after the inhibitor treatments, suggesting the actions of the PP1s on LBR were suppressed in the absence of ELYS (Fig. 4F, siELYS#1). The activities of the PP1s were not reduced by the addition of the CDK and SRPK inhibitors (Fig. 4F, control). Therefore, we concluded that ELYS regulates LBR phosphorylation by supporting the proper action of the PP1s.

To investigate the relationship between the LBR phosphorylation caused by ELYS depletion and the subcellular localization of LBR, we established a HeLa cell line that stably expressed a phosphomimetic (all-A) or unphosphorylated (all-D) mutant of LBR (Fig. 4A). The expression levels of all-D–EGFP and all-A–EGFP were comparable to the expression level of LBR-WT–EGFP but were much lower than that of endogenous LBR (Fig. S3B). The all-D mutant was diffusely localized throughout the nuclear envelope and ER (Fig. 4G; Fig. S3C). These observations support the idea that LBR phosphorylation due to ELYS depletion suppress the nuclear envelope localization of LBR. To confirm the hypothesis in another way, we next examined the localization of LBR in a HeLa cell line that stably expressed the all-A mutant. Rather unexpectedly, the all-A mutant exhibited behavior similar to that of the all-D mutant (Fig. 4G; Fig. S3C). Further complicating the situation, the all-A mutant that had been transiently expressed in HEK293T cells exhibited clear nuclear envelope localization, whereas an all-D mutant lost the ability to localize to the nuclear envelope (Fig. 4H). Note that expression levels of these two constructs were comparable to those in HeLa cells (Fig. S3E).

### LBR phosphorylation facilitates its interaction with lamin B, HP-1 and histone H3, and restricts its mobility in the nuclear envelope

**Fig. 5.**
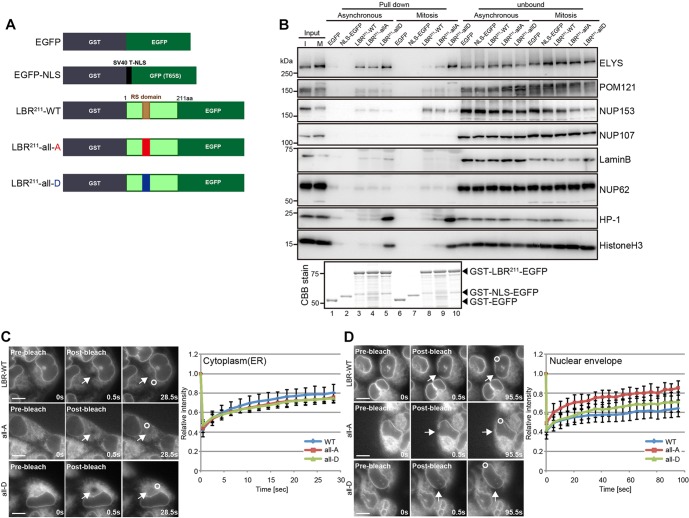
**The constitutively phosphorylated form of LBR interacts with lamin B, HP-1, and histone 3, and its mobility is restricted within the nuclear envelope.** (A) Schematic representation of the GST–LBR^211^–EGFP fragments used in the GST pull-down assay. GST–LBR^211^-all-A–EGFP and GST–LBR^211^-all-D–EGFP both harbor the phosphorylation site mutations described in Fig. 4A. GST–EGFP and GST–NLS–GFP (mutant S65T) were both used as negative controls. (B) The indicated GST–LBR^211^–EGFP-fragment-conjugated GSH beads were incubated with HeLa cell lysates derived from asynchronous (Asynchronous) or nocodazole-arrested cells (Mitosis). The bound proteins were analyzed by western blotting using antibodies against the indicated proteins. The bottom panel represents an SDS-PAGE, 30% of pull-down samples used in western blotting were loaded, stained with Coomassie Brilliant Blue (CBB). I, interphase extract; M, mitotic extract. (C,D) FRAP analysis using the indicated stable LBR–EGFP-expressing cell lines. The intensities of bleached (arrows) and unbleached areas (circle) were measured over the time (see Materials and Methods) – (C) cytoplasm (*n*=9 for LBR-WT; *n*=10 for all-A; and *n*=10 for all-D) and (D) nuclear envelope (*n*=6 for LBR-WT; *n*=6 for all-A; and *n*=7 for all-D). Mean relative values of the bleached area to the unbleached area with the standard deviation for each point were plotted; error bars, s.d.; WT, wild type. Scale bars: 10 µm.

To address the mechanism by which LBR phosphorylation regulates its subcellular localization, we analyzed the biochemical properties of LBR that were influenced by its phosphorylation. We performed a series of pull-down assays from asynchronous or mitotic cell extracts using the N-terminal 211-amino-acid fragment (LBR^211^) of LBR and its mutants fused to GST (Fig. 5A,B). Compared with LBR-WT and the all-A mutant, the all-D mutant interacted with lamin B more strongly in the asynchronous cell extract, whereas this interaction was not observed in the mitotic cell extract. The all-D mutation dramatically enhanced the interaction of LBR with HP-1 and histone H3 in both asynchronous and mitotic cell extracts. Among the Nups, NUP153 and POM121 interacted with all forms of LBR throughout the cell cycle. ELYS strongly interacted with the all-D mutant in mitotic cells, whereas it only weakly interacted with LBR-WT and the all-A mutant. This phosphorylation-dependent interaction was not observed in the asynchronous cell extracts. Taken together, phosphorylation at S71, S86 and at the serine residues in the RS domain of LBR is required for interactions with lamin B in interphase, HP-1 and histone H3 in both interphase and mitosis, and ELYS in mitosis.

All of the phosphorylation-dependent binding partners of LBR identified above reside at the nucleoplasmic face, suggesting the phosphorylation-dependent interactions of LBR regulate its mobility within the nuclear envelope. To assess this, the mobility of LBR–EGFP within the nuclear envelope was examined by fluorescence recovery after photobleaching (FRAP) analyses. Both the unphosphorylated and phosphomimetic mutations had no impact on LBR mobility in the cytoplasm (Fig. 5C), whereas the phosphomimetic mutation restricted LBR mobility within the nuclear envelope relative to the unphosphorylated mutation (Fig. 5D). These results suggest that the phosphorylation of LBR contributes to the mobility of the protein within the nuclear envelope.

### Cytoplasmic interaction between LBR and lamin B is promoted by ELYS depletion

**Fig. 6.**
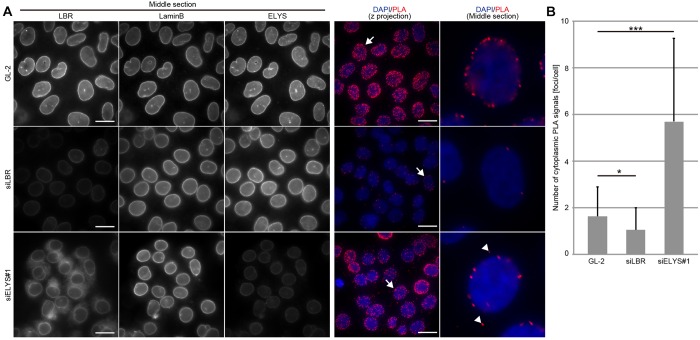
**ELYS depletion facilitates the ectopic interaction of LBR with lamin B.** (A) HeLa cells were transfected with control (GL-2) or siRNA against LBR (siLBR) or siELYS#1 for 48 h and then were used for *in situ* PLA using anti-LBR and anti-lamin-B antibodies. Both the *z*-projected PLA images and enlarged images through the middle section of the nucleus are presented (the two images on the right). The arrows in the *z*-projected PLA images on the far right correspond to the enlarged images of the nucleus middle section. Arrowheads in the enlarged images of the nucleus middle section indicate cytoplasmic PLA signals. The depletion efficiencies of siLBR and siELYS#1 were confirmed by immunostaining (three images on the left). Scale bars: 20 μm. (B) Cytoplasmic PLA signals in control-, siLBR- and siELYS#1-transfected cells were quantified and plotted (*n*=30). The data were analyzed with an unpaired Student's *t*-test; error bars, s.d.; *P<0.05; ***P<0.001.

We examined the interaction between LBR and lamin B in cells using an *in situ* proximity ligation assay (PLA). This technique can detect a protein located adjacent to the protein of interest as a PLA signal. In control cells, most PLA signals were detected at the edge of the nucleus, which was determined by DAPI staining (Fig. 6A, see the middle section of PLA images). Such PLA signals were diminished upon LBR depletion, suggesting that the detected signals are specific to the presence of LBR. In ELYS-depleted cells, PLA signals on the nuclear envelope were largely maintained, indicating that LBR can interact with lamin B on the nuclear envelope in these cells. Additionally, the PLA signals increased in the cytoplasm of ELYS-depleted cells (Fig. 6A,B, see arrowheads in the middle section of PLA image), indicating that ELYS depletion could facilitate the ectopic interaction of LBR with lamin B in the cytoplasm.

### Mislocalization of LBR caused by ELYS depletion can be reversed following CDK and SRPK inhibition

**Fig. 7.**
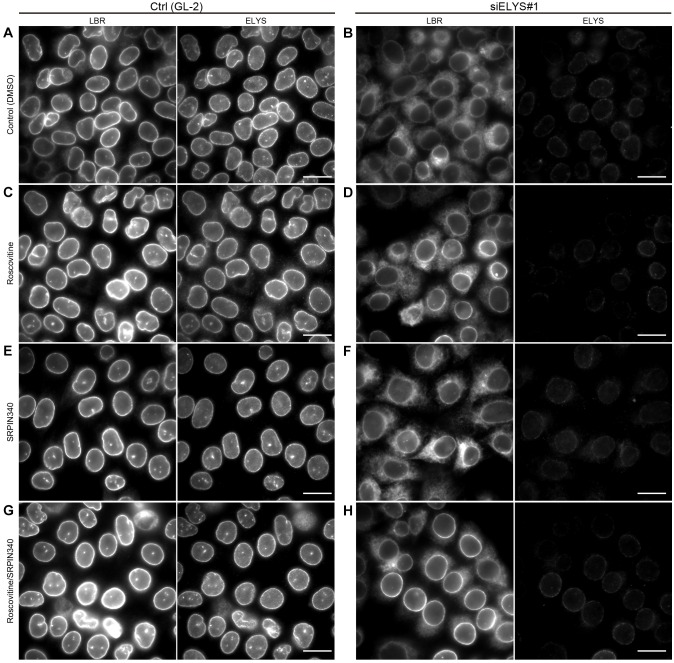
**The localization of LBR in ELYS-depleted cells is restored by treatment with CDK and SRPK inhibitors.** HeLa cells were transfected with the indicated siRNAs (GL-2 or siELYS#1), cultured for 48 h and then treated with the indicated inhibitors for 5 h. The subcellular localization of LBR and ELYS was evaluated by immunostaining. Scale bars: 20 µm.

The negative correlation between LBR phosphorylation and its nuclear envelope localization was indicated by almost all of the observations described above, except for those presented in Fig. 4G. The all-A mutant was not restricted to the nuclear envelope (Fig. 4G), but was phosphorylated at very low levels (Fig. 4C,D). It seems that the localization of LBR in cells cannot simply be explained by its phosphorylation status. To identify additional factor(s) that affect the localization of LBR, we used an alternative approach to re-examine the effects of CDK and SRPKs on LBR mislocalization. For this, ELYS was depleted in HeLa cells by using an siRNA; the cells were then treated with CDK or SRPK inhibitors (Fig. 7). In the control cells, the CDK and SRPK inhibitors had no effect on the nuclear envelope localization of LBR (Fig. 7, control). In the ELYS-depleted cells, the CDK inhibitor partially restored LBR localization to the nuclear envelope (Fig. 7B,D), whereas the SRPK inhibitor did not (Fig. 7B,F). When the ELYS-depleted cells were treated with both inhibitors, LBR localization to the nuclear envelope was largely rescued (Fig. 7H), indicating that the two inhibitors had synergistic effects. Consistent with the results shown in Fig. S2A, the number of NPCs represented by mCherry–NUP107 signals decreased in ELYS-depleted cells, but these signals remained unaffected in the presence of roscovitine and SRPIN340, a condition under which LBR localization to nuclear envelope was rescued (Fig. S4). Importantly, the LBR phosphorylation that was promoted by ELYS depletion was maintained in the cells that had been treated with both kinase inhibitors (Fig. 4F). These results show that additional factors support the nuclear envelope localization of LBR independent of its phosphorylation status and that the activity of those factors is negatively regulated by CDK and SRPKs.

## DISCUSSION

### Phosphorylation has a crucial role in the nuclear envelope localization of LBR

In this study, we proposed that the mislocalization of LBR that is induced by ELYS depletion is primarily caused by increased LBR phosphorylation. NPC number is one of the causative factors for the INM-targeting of transmembrane proteins (Boni et al., 2015; Ungricht et al., 2015). ELYS, NUP107, NUP153 and POM121 are known to be essential for NPC assembly and maintenance (Doucet et al., 2010; Vollmer et al., 2015), and indeed their depletion reduced the density of mAb414-stained foci in our study, although the extent of this reduction varied among the depleted Nups (Fig. S2E). The reductions in the density of mAb414-stained foci in NUP107- (54.6%) and NUP153-depleted cells (57.6%) were higher than those in ELYS- (38.5%) or POM121-depleted cells (22.4%). Under these conditions, the mislocalization and increased phosphorylation of LBR were stronger after ELYS depletion than after NUP107 or NUP153 depletion (compare Fig. 1 and Fig. 2), whereas POM121 depletion, which also weakly reduced the density of mAb414-stained foci, did not affect LBR mislocalization or phosphorylation. Therefore, the mislocalization of LBR seems to be associated with its altered phosphorylation status rather than a reduction in the number of NPCs as defined by the density of mAb414-stained foci. Additionally, inhibiting CDK and SRPKs, which are both kinases responsible for LBR phosphorylation, reverted the nuclear envelope localization of LBR in ELYS-depleted cells without affecting the density of NPCs (Fig.7; Fig. S4). These results agree with our hypothesis that the INM localization of LBR is regulated by phosphorylation signaling that involves CDK and SRPKs.

### Phosphorylation regulation of LBR by CDK, SRPKs and PP1s

We examined the phosphorylation status of LBR by using a Phos-tag western blot, and the obtained results were consistent with previous reports using other methodologies (Courvalin et al., 1992; Takano et al., 2002). LBR is phosphorylated by CDK and SRPKs in interphase (Fig. 3A) at the residues within the consensus sites found in other CDK and SRPK substrates (Fig. 4A–D). Although the phosphatase responsible for LBR dephosphorylation has not yet been conclusively identified (Ito et al., 2007), our observations suggest that PP1 isoforms counteract the activities of CDK and SRPKs to establish the phosphorylation status of LBR.

### ELYS negatively regulates the phosphorylation of LBR, probably by modulating the action of PP1s

We found that ELYS depletion in interphase cells promotes LBR phosphorylation (Fig. 1E,F). Under steady-state conditions, ELYS could either repress the actions of CDK and SRPKs or support the action of PP1s on LBR. Considering the results shown in Fig. 4F, in which the highly phosphorylated form of LBR observed in the ELYS-depleted cells was stably maintained after further treatment with CDK and SRPK inhibitors, we favor the latter idea.

Assuming that ELYS regulates the action of PP1s on LBR, one might question how this regulation is accomplished. The properties of PP1 isoforms, such as subcellular localization, substrate affinity and total activity, might be dictated by ELYS in a manner similar to how these properties are dictated by their diverse binding partners (Bollen et al., 2010). However, regarding the subcellular localization of PP1α and PP1γ, there was no global alteration upon ELYS depletion (data not shown). The interaction between ELYS and LBR during interphase (Fig. 5B) might account for the actions of the PP1s on LBR. We showed that LBR phosphorylation was also increased upon the depletion of other Nups, such as NUP107 and NUP153 (Fig. 2D). Interestingly, previous reports have identified NUP153 as a binding partner of PP1s (Liu et al., 2010; Moorhead et al., 2008), and both ELYS and NUP153 constantly interact with the NUP107-160 subcomplex in interphase (Rasala et al., 2006; Vasu et al., 2001). These situations give rise to the possibility that the whole NPC, and not ELYS alone, could enable the efficient dephosphorylation of LBR by recruiting both LBR and PP1s and bringing them near to each other. We could not detect an interaction between endogenous LBR and endogenous PP1γ1 fused with mClover using an *in situ* PLA with antibodies against LBR and GFP antibodies (data not shown). Clearly, further work is required to understand the mechanism of LBR dephosphorylation.

### A phosphomimetic mutation restricts the mobility of LBR within the nuclear envelope through its phosphorylation-dependent interactions but impedes its nuclear envelope localization

The phosphomimetic mutation restricted the mobility of LBR within the nuclear envelope relative to the non-phosphorylatable mutation (Fig. 5C,D), suggesting that LBR phosphorylation positively regulates its nuclear envelope retention. This idea was supported by our biochemical observations showing the phosphorylation-dependent interaction of LBR with its binding partners (Fig. 5B). Seemingly contradictorily, the phosphomimetic LBR mutant failed to localize efficiently to the nuclear envelope (Fig. 4G,H). Considering that the nuclear envelope localization of LBR is achieved in two steps (nuclear envelope targeting and nuclear envelope retention), the LBR phosphorylation might negatively regulate the former step but regulate nuclear envelope retention positively. Alternatively, LBR can generate cytoplasmic PLA signals with lamin B following ELYS depletion (Fig. 6), raising the possibility that aberrantly phosphorylated LBR, which was dispersed throughout the ER, ectopically interacts with lamin B in the cytoplasm and that this ectopic interaction might inhibit the nuclear-envelope-targeting step of LBR. To further investigate this possibility, it will be important to analyze the ectopic interactions of LBR using the other binding partners of LBR, including histone 3 and HP1, whose interactions are enhanced by phosphorylation of LBR.

The localization of the non-phosphorylatable mutant of LBR showed cell-type specific differences; it was dispersed throughout the ER in HeLa cells and restricted to the nuclear envelope in HEK293T cells (Fig. 4G,H). A previous report has shown that preferential interaction with histones that have specific modifications is crucial for the nuclear envelope localization of LBR (Hirano et al., 2012). As exemplified there, the observed cell-type specific differences might be explained by cell-type specific differences in the biochemical properties of LBR-binding partners.

In this study, we showed that ELYS regulates the nuclear envelope localization of LBR by altering the balanced actions of two types of kinases, CDK and SRPKs, and the PP1 phosphatases. When these balanced actions on LBR were impaired by ELYS depletion, LBR became over phosphorylated and mislocalized. The depletion of other Nups, such as NUP107 and NUP153, caused a similar phenotype. These observations imply the existence of an NPC-governed phosphorylation network that regulates the nuclear envelope localization of LBR. To evaluate the general significance of such a network, other factors subjected to the same phosphorylation network must be identified.

## MATERIALS AND METHODS

### Cell culture, transfection and cell cycle synchronization

HeLa and HEK293T cells were cultured in Dulbecco's modified Eagle's medium (Life Technologies) supplemented with 10% fetal bovine serum (FBS; Sigma-Aldrich) at 37°C under 5% CO_2_.

In Fig. 1C,D and Fig. 5B, HeLa and HEK293T cells were synchronized at prometaphase by treatment with 100 ng/ml nocodazole (16 h) (Sigma-Aldrich) and then harvested by shaking. In Fig. 1E, the HeLa cells that had been transfected with the siRNAs were first synchronized at G1/S phase with 2 mM thymidine (T1895, Sigma) treatment (16 h), released and cultured for 8 h, and then further cultured in medium containing 100 ng/ml nocodazole for 4 h. The mitotically arrested cells were harvested by shaking.

### Plasmid and siRNA transfections

The plasmid and siRNA transfections were performed using FugeneHD (Promega Corporation) and Lipofectamine RNAiMAX (Life Technologies), respectively. The siRNA oligonucleotides are described in Table S2.

### Plasmid construction

LBR cDNA was obtained as previously described (Funakoshi et al., 2011). To generate pEGFP-N3-LBR-WT and pEGFP-N2-LBR^211^, full-length LBR cDNA and LBR^211^ fragment were amplified by PCR using pFRT-V5-P_EF1α_-LBR-EGFP (Clever et al., 2012) as a template and subcloned into the pEGFP-N3 vector or the pEGFP-N2 vector (Clontech Laboratories).

The LBR^211^-EGFP and EGFP fragments were digested from pEGFP-N2-LBR^211^ and pEGFP-N2, respectively. These fragments were inserted into the pGEX-6P-1 vector (GE Healthcare) to generate pGEX-6P-1-LBR^211^-EGFP and pGEX-6P-1-EGFP, which were used to express the recombinant proteins (Fig. 5A). pGEX-2T-NLS-GFP(T65S) is subcloned GFP(T65S) fused with the SV40T NLS sequence (PPKKKRKVEDP) at its N-terminus into the pGEX-2T vector and was kindly provided by Dr Yoneda (National Institute of Biomedical Innovation, Osaka, Japan). To generate pGEX-6P-1-NLS-GFP (T65S), the NLS-GFP fragment was excised from pGEX-2T-NLS-GFP (T65S) and inserted into the pGEX-6P-1 vector.

pEXPR-P_EF1α_-LBR-EGFP was constructed using the multi-site Gateway system, as previously described (Sasaki et al., 2004). To establish a stable mCherry–NLS-expressing HeLa cell line (Fig. S1G), pmCherry-C1-T-NLS was constructed by inserting the SV40T-NLS sequence (PPKKKRKVEDP) between the BamHI and XbaI sites in the pmCherry-C1 vector (Clontech).

Both the unphosphorylated and phosphomimetic LBR mutants (Fig. 4A) were generated using the KOD-Plus-mutagenesis kit (TOYOBO, Japan).

All of the primers used in this study are listed in Table S1.

### Inhibitors

To inhibit CDK activity, roscovitine (R7772, Sigma-Aldrich) was added to the culture medium at a final concentration of 40 µM. To inhibit SRPK1 and SRPK2 activity, SRPIN340 (504293, Millipore) was added to the culture medium at a final concentration of 50 µM.

### Establishment of stable cell lines

Cell lines stably expressing LBR-WT–EGFP, the LBR all-A mutant and LBR all-D mutant were obtained as previously described (Yahata et al., 2005), using Effectene (Qiagen). Cells that stably expressed mCherry–NLS were established through selection with 700 µg/ml geneticin (Roche, Switzerland).

### Live-cell imaging and FRAP assay

For live-cell imaging and the FRAP assay, cells were grown in a 3.5-cm glass-bottom dish (Iwaki, Japan) in DMEM without Phenol Red (Life Technologies) and supplemented with 10% FBS, and then observed under an inverted microscope (IX-71 DeltaVision CORE system; Olympus and Applied Precision, Issaquah, WA) in a humidified environmental chamber (MI-IBC, Olympus, Japan) maintained at 37°C and under 5% CO_2_. The images were captured from a single focal plane with a 60×1.40 Plan Apo objective lens (Olympus) and a Cool Snap HQ2 CCD camera (Photometrics Inc., Tucson, AZ).

Photobleaching of the cytoplasm (Fig. 5C) or the nuclear envelope (Fig. 5D) was performed with a DeltaVision microscopy system equipped with a quantifiable laser module (50 mV, 488-nm solid-state laser). After pre-bleaching, images were acquired with a 60×1.40 Plan Apo objective lens; the cytoplasm or the nuclear envelope in the region of interest was then bleached with a 1-s stationary pulse at 100% laser power. Images were acquired immediately after bleaching, and subsequent images were captured every 2 s (Fig. 5C) or 5 s (Fig. 5D). The fluorescence intensity in the bleached and unbleached areas of the cytoplasm (Fig. 5C) or the nuclear envelope (Fig. 5D) was quantified in images using SoftWorx software (Applied Precision) with a circle that was 19 pixels in diameter, as previously described (Funakoshi et al., 2011). The relative intensities (bleached area to unbleached area) were calculated from the measured total intensities, from which the intensity determined outside the cell was subtracted, and then normalized to the relative intensity in the pre-bleaching image.

### Immunostaining

Cells were grown on poly-L-lysine (Wako, Osaka, Japan)-coated coverslips and fixed with 2% paraformaldehyde (Wako) in PBS for 10 min. The fixed cells were permeabilized with 0.5% Triton X-100 in PBS for 5 min and blocked with 5% normal goat serum (NGS; Chemicon, Temecula, CA) in PBS for 1 h. Cells were stained with primary antibodies (1% NGS in PBS) for 2 h and secondary antibodies (1% NGS in PBS) for 2 h, and then counterstained with DAPI (Roche) and mounted in PPDI [80% glycerol in PBS, 1 mg/ml paraphenylenediamine (11873580001, Roche)]. Images were recorded with a DeltaVision microscope using 60×1.40 and 100×1.35 Plan Apo objective lenses. For lamin-B staining, 5% bovine serum albumin (BSA, Sigma-Aldrich) in PBS was used as blocking buffer, and 1% BSA in PBS was used for dilution of antibodies (Fig. S1B).

### Western blotting with or without Phos-tag

For western blotting, cells that had been lysed in 2× Laemmli sample buffer (125 mM Tris-HCl, pH 6.8, 20% w/v glycerol, 4% w/v SDS, 200 mM DTT and 0.01% w/v Bromophenol Blue) were subjected to SDS-PAGE and blotted onto a PVDF membrane (IPVH00010, Millipore). The membranes were blocked in 0.2% w/v casein in TBS containing 0.05–0.1% Tween-20 (TBS-T) and probed with primary and secondary antibodies diluted in 0.2% w/v casein in TBS-T.

For Mn^2+^-Phos-tag western blotting, the cells were washed in Hepes-NaOH buffer (20 mM Hepes, pH 7.4, and 137 mM NaCl) twice and lysed with 2× Laemmli sample buffer. The lysate was electrophoresed on a separate SDS-PAGE gel containing 25 µM Phos-tag (AAL-107 M, Wako) and 50 µM MnCl_2_ (Kinoshita-Kikuta et al., 2007). To inactivate the Phos-tag after electrophoresis, the gel was incubated in inactivation buffer (25 mM Tris, 5% v/v methanol and 10 mM EDTA) for 10 min and further incubated in inactivation buffer without EDTA for 10 min. The proteins were then blotted onto a PVDF membrane and blocked with 1% v/v gelatin in TBS-T; the LBR protein bands were detected with specific antibodies.

### Quantification of signals in western blotting and immunostaining

To quantify fluorescence intensities at the nuclear envelope, fluorescent signals were measured using SoftWorx software, as described previously (Maeshima et al., 2010) with minor modifications. The fluorescence intensity of LBR or LBR–EGFP at the nuclear envelope was extracted using a circle 19 pixels in diameter that comprised the nuclear envelope in the center. The intensity of cytoplasm staining was measured with a circle 19 pixels in diameter. The measured total intensities of the nuclear envelope and cytoplasm, from which the background obtained from outside of the cell was subtracted, were used to calculate the nuclear envelope to cytoplasm ratio (Figs 1A,B, 2A–C, 3E–F and 4G). The line profiles in Figs S1A and S3C were constructed using SoftWorx software.

The signal intensities in western blot images were measured with ImageJ software. The measured signal intensity, from which the background signal obtained from an area outside of the measured signal in the same lane was subtracted, was normalized to the β-actin signal (Fig. S5A and C) or the total intensities of LBR bands (low-, high- and hyper-phosphorylated species) in the Phos-tag western blot (Figs 1C,E,F, 2D, 3A–D and 4E,F). To calculate the depletion efficiency of the target protein in the siRNA-transfected cells described in Figs 1D–F, 2D and 3B, the signal intensities of the target protein, which were normalized to the β-actin signal, were calculated in control and siRNA-transfected cells. The normalized value in siRNA-transfected cells was divided by that in control-siRNA-transfected cells.

### Protein purification and GST pull-down assay

LBR^211^–EGFP, LBR^211^-all-A–EGFP, LBR^211^-all-D–EGFP, EGFP–NLS or EGFP was expressed in BL-21 bacteria. The recombinant proteins were induced by the addition of 0.1 mM isopropyl β-D-1-thiogalactopyranoside (IPTG) (Nacalai Tesque), followed by 3 h of culture at 37°C. The cells were harvested, lysed by sonication in lysis buffer (50 mM Tris-HCl, pH 7.4, 250 mM NaCl, 0.1% Triton X-100, 1 mM EDTA, 1 mM DTT and 0.3 mM PMSF) and clarified (21,500 ***g***, 20 min). The recombinant proteins were purified from the clarified lysate with glutathione Sepharose04B beads (GSH beads, GE Healthcare) according to the manufacturer's instructions. A HeLa cell lysate was prepared as reported previously (Hawryluk-Gara et al., 2005). Briefly, asynchronous or nocodazole-arrested HeLa cells were lysed with lysis buffer A [10 mM Tris-HCl, pH 7.4, 400 mM NaCl, 1% Triton X-100, 2 mM EDTA, 1 mM DTT, complete proteinase inhibitor cocktail (Roche) and PhosSTOP (Roche)], sonicated, incubated for 30 min at 4°C and clarified (21,500 ***g***, 20 min at 4°C). The clarified supernatant was diluted 3.75-fold with dilution buffer (10 mM Tris-HCl, pH 7.4, 2 mM EDTA, 1 mM DTT, complete proteinase inhibitor cocktail and PhosSTOP), added to GSH beads that had been conjugated with GST proteins and incubated for 1 h at 4°C. The bound proteins were analyzed by western blotting.

### *In situ* PLA

An *in situ* PLA was conducted using the Duolink system (Sigma) according to the manufacturer's instructions. Two sets of control, LBR-siRNA transfected and ELYS-siRNA-transfected HeLa cells were prepared. One set was co-immunostained with antibodies against LBR and lamin B, and the other set was used for *in situ* PLA with the same primary antibodies used in co-immunostaining. Images of PLA signals were acquired from 15 sections with 0.5-µm intervals using a DeltaVision microscope with a 60×1.40 Plan Apo objective lens for acquisition of the immunostained images, and are shown as their maximum intensity projections, generated by using SoftWorx software. The quantification of *in situ* PLA signals was performed by manual counting based on DAPI staining and differential interference contrast images.

### Antibodies

The primary antibodies used for immunofluorescence staining were mouse anti-ELYS (BMR00513, BioMatrix Research), 1:500; rabbit anti-LBR (1398-1, Epitomics), 1:1000; rabbit anti-emerin (sc-15378, Santa Cruz Biotechnology), 1:1000; goat anti-lamin-B (sc-6217, Santa Cruz Biotechnology), 1:200; mouse anti-lamin-A/C (sc-7292, Santa Cruz, Biotechnology), 1:1000; rabbit anti-Lap2α (IQ175, ImmuQuest), 1:250; mouse anti-Lap2β (611000, BD Transduction Laboratories), 1:1000; mAb414 (MMS-120P, Covance), 1:3000; rat anti-NUP153 (R4C8, BioAcademia, Osaka, Japan), 1:3000; rat anti-POM121 (Funakoshi et al., 2011), 1:1000; and mouse anti-calnexin (ab31290, Abcam), 1:1000 antibodies. The secondary antibodies used for immunofluorescence were donkey anti-rabbit Alexa-Fluor-488 (A21206), anti-mouse Alexa-Fluor-647, goat anti-mouse Alexa-Fluor-594 (A11032) and donkey anti-goat Alexa-Fluor-594 (A11058); all secondary antibodies were purchased from Molecular Probes (Eugene, OR) and used at a dilution of 1:800.

The primary antibodies used for western blotting were the same as those used in immunofluorescence and were used at the same dilutions, with the additional antibodies: mouse anti-phosphorylated-histone-H3 at residue S10 (6G3, Cell Signaling Technology, MA), 1:2000; mouse anti-β-actin (A5441, Sigma-Aldrich), 1:3000; rabbit anti-GFP (598, MBL, Nagoya, Japan), 1:1000; mouse anti-GFP (11-814-460-001, Roche), 1:1000; goat anti-GFP (AB0020-200, SICGEN antibodies, Cantanhede, Portugal), 1:1000-2000; anti-PP1α (sc-6104, Santa Cruz Biotechnology), 1:2000; anti-PP1β (sc-6107, Santa Cruz Biotechnology), 1:2000; anti-PP1γ (sc-6109, Santa Cruz Biotechnology), 1:10,000; rabbit anti-NUP107 (A301-787A, Bethyl Laboratories, Montgomery, TX), 1:1000; rabbit anti-HP-1 (C7F11, Cell Signaling Technology), 1:500; and rabbit anti-histone-3 (ab1791, Abcam), 1:4000. The mAb414 antibody was used (MMS-120P, Covance) to detect NUP62 at a 1:3000 dilution. The secondary antibodies used for western blotting were: goat anti-mouse horseradish peroxidase (HRP) (170-6516, Bio-Rad, Hercules, CA), 1:3000; goat anti-rabbit-HRP (170-6515, Bio-Rad), 1:3000; and rabbit anti-goat HRP (JIR305-035-003, Jackson ImmunoResearch, West Grove, PA), 1:2000.
